# LIVED EXPERIENCE OF DEMENTIA FAMILY CAREGIVERS IN RURAL CHINA: FINDINGS FROM A QUALITATIVE STUDY

**DOI:** 10.1093/geroni/igae098.4232

**Published:** 2024-12-31

**Authors:** Dexia Kong

**Affiliations:** The Chinese University of Hong Kong, Hong Kong, China (People’s Republic)

## Abstract

**Background:**

Currently, China has the highest prevalence of dementia worldwide. The prevalence of dementia is greater in rural areas than in urban ones, mostly because over two-thirds of Chinese older adults reside in rural regions. Moreover, the cultural preference for family-based eldercare, in conjunction with the absence of dementia-related services in rural areas of China, has resulted in the responsibility of caring for older adults with dementia falling solely on family caregivers. Nevertheless, there has been no prior research that has examined the lived experiences of caregivers of individuals with dementia in rural China. This study investigates the lived experiences of dementia family caregivers in rural China, thereby addressing the knowledge gap.

**Methods:**

In-depth qualitative data were collected from 25 family caregivers residing in a rural county in southwestern China. All interviews were recorded and transcribed verbatim. The interview narratives were analyzed using the conventional content analysis approach. All transcripts were coded by two experienced researchers with qualitative training. Discrepancies in coding were resolved by consensus.

**Results:**

Main themes that emerged from the data include caregivers’ perceptions of dementia, motivation for caregiving, challenges in caregiving, dynamics within family relationships, coping strategies, and unmet needs.

**Conclusion:**

In rural China, families are crucial to the care of older adults living with dementia. The findings underscore the pressing need to develop psychoeducational intervention strategies for supporting family caregivers of individuals with dementia in rural or low-resource regions in China. Policy and service implications will also be discussed.

